# Frequency of orthostatic hypotension in the Pooled Resource Open-Access ALS Clinical Trials database

**DOI:** 10.3389/fneur.2025.1512357

**Published:** 2025-01-16

**Authors:** C. Quarracino, F. Capani, M. Otero-Losada, G. E. Rodríguez, S. Pérez-Lloret

**Affiliations:** ^1^Centro de Altos Estudios en Ciencias Humanas y de la Salud, Universidad Abierta Interamericana, Consejo Nacional de Investigaciones Científicas y Técnicas, CAECIHS, UAI-CONICET, Buenos Aires, Argentina; ^2^Instituto de Ciencias Biomédicas, Facultad de Ciencias de la Salud, Universidad Autónoma de Chile, Santiago, Chile; ^3^Clínica Enfermedades de Neurona Motora, División Neurología, Hospital General de Agudos José María Ramos Mejía, Buenos Aires, Argentina; ^4^Instituto Universitario de Ciencias de la Salud, Fundación H.A. Barceló, Consejo Nacional de Investigaciones Científicas y Técnicas (CONICET), Buenos Aires, Argentina; ^5^Departamento de Fisiología, Facultad de Medicina, Universidad de Buenos Aires (UBA), Buenos Aires, Argentina

**Keywords:** ALS dysautonomia, cardiac dysautonomia, orthostatic hypotension, sympathetic cardiac dysautonomia, amyotrophic lateral sclerosis

## Abstract

**Purpose:**

To explore the frequency of orthostatic hypotension (OH) in a large sample of amyotrophic lateral sclerosis patients (ALS).

**Methods:**

From the PRO-ACT database, data of 1,240 ALS patients were analyzed, focusing on blood pressure and heart rate before and after standing. OH was defined as a drop in systolic/diastolic blood pressure > 20/10 mm Hg within 3 min of standing. Neurogenic OH was diagnosed when the heart rate increase was below 15 bpm in patients not taking medications that could affect this response.

**Results:**

At baseline, 138 (11.1%) patients showed OH, 76.1% of whom had neurogenic OH. At follow-up, 163 patients (13.1%) had OH, 71.2% with neurogenic OH. Only 22.5% of the patients with OH at baseline had OH at follow-up.

**Conclusion:**

In a large sample of ALS patients, OH occurred in 11–13%, pointing to a subgroup that might require special care to avoid related complications.

## Introduction

Amyotrophic lateral sclerosis (ALS) is a neurodegenerative disease classically believed restricted to the motor pathway (1, 2). In more prevalent neurodegenerative diseases like Parkinson’s (PD), non-motor symptoms have gained attention and are associated with increased morbidity and mortality (3, 4).

The overlap between cognitive dysfunction and ALS has been known for several years (5). Yet, there is no consensus about the concurrence of sensitive or autonomic dysfunction. Among dysautonomia symptoms, orthostatic hypotension (OH) is of the utmost importance since its role as a risk factor of myocardial infarction, stroke, vascular disease, and even death (6, 7). It can be classified as non-neurogenic (secondary to impaired vasoconstriction or reduced cardiac function) or neurogenic (determined by a pathologic autonomic response). The latter is defined by an inadequate heart rate response in the context of OH (6, 8).

Several studies employing small samples have suggested OH is infrequent in ALS (9–12). However, this issue has not been explored seemingly in larger samples. The Pooled Resource Open-Access ALS Clinical Trials database (PRO-ACT) is an ALS patient repository that includes de-identified records of over 8,500 individuals from 17 phase II and III clinical trials held between 1990 and 2010 (13).

We aimed to address the frequency of OH in a large sample of ALS patients.

## Method

We selected ALS patients with two measurements of blood pressure and heart frequency variation under orthostatic stress from the 10,723 records of the PRO-ACT database. OH was defined as the fall in systolic and/or diastolic blood pressure > 20/10 mm Hg within the first 3 min after standing up (8, 14). Neurogenic OH was considered when: (1) OH was present; (2) no medications were being administered for Parkinson’s disease or diabetes or drugs that could prevent a compensatory heart rate increase upon standing such as adrenergic alpha- or beta-blockers, non-dihydropyridine calcium channel blockers, central alpha-2 agonists, or amiodarone (14), and (3) heart rate increase was lower than 15 bpm after standing up (6, 14). In patients with OH with a systolic component, the ratio between heart rate change and systolic blood pressure drop (HR/SBP ratio) was determined, as values below 0.492 are diagnostic of neurogenic origin (15). Blood pressure was measured twice, separated by a median of 8.8 months.

The frequency of OH was compared with that of a control group of healthy individuals of similar sex and age from a different database (16).

The t-test or Pearson chi-square test was used for between-group bivariate comparisons and the McNemar’s test was used for dependent samples comparison.

## Results

Out of 1,240 eligible records, 779 (62.8%) were of men, and 1,161 (95%) were of white race, with 54.07 ± 12.28 years old mean age ± SD. Data of time since symptom onset, form of onset, ALS-FRS, comorbidities, and forced vital capacity (FVC) was missing.

None of the patients were being pharmacologically treated for OH, including midodrine, droxidopa, fludrocortisone, pyridostigmine, or desmopressin, as per the analysis of the PRO-ACT medication records. One thousand fourteen patients (81.8%) were using at least one medication associated with OH, such as antihypertensive drugs, diuretics, antidepressants, dopaminergic agents, anticholinergics, vasodilators, alpha-2 agonists, antipsychotics, and benzodiazepines. Eleven patients (0.9%) were under treatment with dopaminergic or sugar-lowering drugs, while 166 (13.4%) were receiving drugs that might affect the heart rate response during orthostatic stress.

At baseline (Table 1), 138 patients (11.1%) had OH. Most of them (*n* = 105, 76.1%) had neurogenic OH. The mean ± SD heart rate, systolic blood pressure (SBP) and diastolic blood pressure (DBP) differences (bpm, standing minus decubitus) in patients with OH were 4.46 ± 11.00, −14.43 ± 13.31, and − 9.93 ± 8.38, respectively. Of the 138 patients with OH, 45 (32%) fulfilled SBP criteria, 70 (51%) DBP criteria, and 23 (17%) both. Among those with SBP or SBP + DBP criteria for OH diagnosis (*n* = 68), 89% had an HR/SBP ratio below 0.492. Systolic/diastolic blood pressure (SBP/DBP) and heart rate (beats per minute) in the supine position were, respectively, 130.8 ± 16.4, 82.0 ± 10.4, and 74.1 ± 10.8; and 131.9 ± 17.8, 83.5 ± 11.3, and 79.8 ± 13.1 in the standing position. Three-hundred forty-one patients (27.5%) had supine SBP/DBP values >140/90 mm Hg and thus were considered as hypertensives. The frequency of hypertension among the patients without OH, with non-neurogenic OH, or with neurogenic OH was 26.2, 14.3, and 40.0%, respectively. The chi-sq test revealed a non-significant difference in the prevalence of HTA between patients without OH and those with non-neurogenic OH (*p* = 0.48), while the difference between the former and the patients with neurogenic OH reached statistical significance (*p* = 0.005).

**Table 1:** Orthostatic hypotension assessment at baseline and after the follow-up period in ALS patients.

	Follow-up		
	No OH (*N* = 1,077)	OH neurogenic (*N* = 116)	OH non-neurogenic (*N* = 47)
Baseline			
No OH (*N* = 1,102)	970 (90.1%)	96 (82.8%)	36 (76.6%)
OH neurogenic (*N* = 105)	86 (8.0%)	16 (13.8%)	3 (6.4%)
OH non-neurogenic (*N* = 33)	21 (1.9%)	4 (3.4%)	8 (17.0%)

ALS, amyotrophic lateral sclerosis; OH, orthostatic hypotension.

There were no statistically significant differences in sex (*p* = 0.392), age (*p* = 0.261), race (*p* = 0.584), or use of OH-associated medications (*p* = 0.128) between patients with and without baseline OH. Patients with OH with or without neurogenic characteristics were not different according to sex (*p* = 0.805), age (*p* = 0.086), or race (*p* = 0.117). However, patients with neurogenic OH were less likely to be taking OH-associated drugs than those with non-neurogenic OH (70% vs. 97%, *p* = 0.002).

At follow-up (Table 1), 163 patients (13.1%) had OH and 116 patients (71.2%) had neurogenic OH. The mean ± SD heart rate, SBP and DBP differences (bpm, standing minus decubitus) in patients with OH were 5.64 ± 11.83, −14.70 ± 13.94 and − 9.22 ± 9.14, respectively. Of the 138 patients with OH, 55 (34%) fulfilled SBP criteria, 82 (50%) DBP criteria and 26 (16%) both. Among those with SBP or SBP + DBP criteria for OH diagnosis (*n* = 81), 64% had an HR/SBP ratio below 0.492. Supine SBP/DBP (mm Hg) and heart rate (bpm) in the standing position were 131.0 ± 17.3/81.5 ± 10.5, and 76.8 ± 10.8 and 131.5 ± 17.7/83.3 ± 11.1 and 81.8 ± 13.8. There were no differences with the respective baseline values in supine or standing SBP/DBP and heart rate (*p* = 0.73, *p* = 0.15, *p* = 0.45, *p* = 0.65). However, heart rate frequency in the supine and standing positions increased by about 3 bpm compared with baseline (*p*-values <0.01).

Patients with and without OH were not statistically different according to sex (*p* = 0.963), race (*p* = 0.244), or use of medications associated with OH (*p* = 0.555), while the former ones were older than the latter ones (55.97 ± 11.59 vs. 53.78 ± 12.36 years old, *p* = 0.034). There were no differences in sex (*p* = 0.833), age (*p* = 0.518), or race (*p* = 0.412) among patients with or without neurogenic OH. Anew, among patients with OH, those with neurogenic OH were less frequently on OH-associated drugs (77% vs. 100%, *p* < 0.001).

Compared against 108 healthy individuals (56.5% men, mean age 57.73 ± 11.62 years), of whom 6 (5.6%) had OH, the frequency of OH was the same at baseline (*p* = 0.07) but different at follow-up (*p* = 0.02).

Regarding OH during both evaluations, most patients (77.5%) with baseline OH, both neurogenic and non-neurogenic, had no OH at follow-up (Table 1), and only 22.5% (*n* = 31) had persistent OH. Among them, 12 (39%) fulfilled SBP criteria, 11 (35%) DBP criteria and 8 (26%) both. Although the small sample size limits the statistical analysis, there seems to be a trend in patients fulfilling DBP criteria with sustained OH. Neither the frequency variation of OH vs. no OH nor neurogenic vs. non-neurogenic OH between baseline and follow-up reached statistical significance using the McNemar test (*p* = 0.121 and *p* = 0.999, respectively) for paired samples.

## Discussion

The mean OH frequency at both measurements was around 12%, higher than the 6–10% prevalence reported in the middle-aged population (7, 17–19). Arguably, ALS patients are more prone to physical deconditioning due to impaired mobility and taking medications associated with OH, like amitriptyline, muscle relaxants, or antidepressants. Yet, most patients had a pathological heart rate response to OH, suggesting dysautonomia, unrelated to their ambulatory capacity or use of medications associated with OH, the latter evenly distributed among patients with and without OH.

The frequency of neurogenic OH was lower than described for other neurodegenerative diseases with dysautonomia like PD (50%), Lewy body dementia (50%), multiple system atrophy (70%), and pure autonomic failure (100%) (6, 8, 20), yet higher than the 5.6% observed in a sample of age- and gender-matched healthy controls (16). Oxidative stress is one of the environmental factors associated with ALS (1, 2) and, eventually, dysautonomia (21). Hence, these findings might put towards a subgroup of patients with a particular phenotype. Further research is necessary to explore this hypothesis.

While previous studies have failed to detect OH in this disease (9–12, 22, 23), some have observed an altered adrenergic behavior (9, 11). Noteworthy, most of them included samples with less than 60 patients, which could account for their negative results.

Non-neurogenic-OH was associated with the more frequent use of OH-associated medications, already described as a risk factor for OH (6, 8, 24). Yet, as patients with and without OH showed no differences, we believe this finding can be sample-related.

Strikingly, only 22.5% of patients having baseline OH showed a pathologic response to orthostatic stress at follow-up. A similar finding and proportion (20%) were described in a larger sample of middle-aged men (*n* = 5,722) in the Malmö Preventive Project (25) and attributed to the known daily and seasonal blood pressure variability and low reliability of a single BP measurement after standing compared with a continuous tilt-test blood pressure monitoring (25, 26). Yet, the OH definition (8, 14) considers manual measurement valid. Hence, even if prone to error, our analysis shows a higher frequency of OH compared with an age- and gender-matched sample of healthy controls (16). However, we believe it important to highlight that, as observed by Fedorowski et al. (25), patients with sustained OH are at higher risk of cardiovascular events, making their identification of vital importance.

Our analysis has several limitations. The source database includes 10,723 patients and selection bias might be present. Moreover, there was no register of comorbidities known as associated with neurogenic OH, like diabetes and Parkinson’s disease. Nevertheless, fewer than 1% of the patients were on medications for any of those conditions. Since information regarding disease severity (ALS-FRS or FVC) was missing, determining the motor functionality stage corresponding to our findings was not possible. However, considering that the PROACT database is made of records of phase II and III trials that usually enroll patients within the first 2 years after symptom onset, our findings are unlikely to correspond with severe morbidity. Also, there was no information on OH symptoms or related outcomes, like falls, fall-related injury or death, target-organ damage, etc., to enrich OH characterization in ALS and help guide future research. Multiple blood pressure measurements after standing could have increased assessment reliability. Finally, data on sex, age, disease severity, race, time since symptom onset, form of disease onset, comorbidities, and FVC were not in the database and could not be analyzed. Further studies are needed to better understand their impact on OH in ALS.

This appears to be the largest ALS patient sample study that evaluates OH. Noteworthy, its frequency was over 10%, showing a subgroup of ALS patients that might require special care to avoid related complications. Well-designed studies are necessary to confirm these findings.

## Data Availability

The datasets presented in this study can be found in online repositories. The names of the repository/repositories and accession number(s) can be found at: https://ncri1.partners.org/proact.

## References

[1] Gordon PH. Amyotrophic lateral sclerosis: pathophysiology, diagnosis and management. CNS Drugs [Internet]. (2011) 25:1–15. doi: 10.2165/11586000-000000000-00000 21128691

[2] Morgan S Orrell RW. Pathogenesis of amyotrophic lateral sclerosis. Br Med Bull [Internet]. (2016) 119:87–98. doi: 10.1093/bmb/ldw026 27450455

[3] Macphee GJA MacMahon DG. Non-motor symptoms and co-morbidity in Parkinson’s disease. En: Chaudhuri KR Tolosa E AHV Schapira Poewe W, editors. Non-motor symptoms of Parkinson’s disease [internet]. Oxford: Oxford University Press; (2014)

[4] Petersen JD Waldorff FB Siersma VD Phung TKT Bebe ACKM Waldemar G. Major depressive symptoms increase 3-year mortality rate in patients with mild dementia. Int J Alzheimers Dis [Internet]. (2017) 2017:1–8. doi: 10.1155/2017/7482094 PMC5397625 28484660

[5] Ferrari R Kapogiannis D Huey ED Momeni P. FTD and ALS: a tale of two diseases. Curr Alzheimer Res [Internet]. 8:273–94. doi: 10.2174/156720511795563700 PMC3801195 21222600

[6] Kalra DK Raina A Sohal S. Neurogenic orthostatic hypotension: state of the art and therapeutic strategies. Clin Med Insights Cardiol. (2020) 14:117954682095341. doi: 10.1177/1179546820953415 PMC7466888 32943966

[7] Ricci F De Caterina R Fedorowski A. Orthostatic hypotension. J Am Coll Cardiol [Internet]. (2023) 66:848–60. doi: 10.1016/j.jacc.2015.06.1084 26271068

[8] Palma JA Kaufmann H. Management of orthostatic hypotension. Continuum [Internet]. (2022) 26:154–77. doi: 10.1212/CON.0000000000000816 PMC7339914 31996627

[9] Oprisan AL Popescu BO. Dysautonomia in amyotrophic lateral sclerosis. Int J Mol Sci. (2023) 24:14927. doi: 10.3390/ijms241914927 37834374 PMC10573406

[10] Pavlovic S Stevic Z Milovanovic B Milicic B Rakocevic-Stojanovic V Lavrnic D. Impairment of cardiac autonomic control in patients with amyotrophic lateral sclerosis. Amyotroph Lateral Scler [Internet]. (2010) 11:272–6. doi: 10.3109/17482960903390855 20001491

[11] Linden D Diehl RR Berlit P. Reduced baroreflex sensitivity and cardiorespiratory transfer in amyotrophic lateral sclerosis. Electroencephalogr Clin Neurophysiol [Internet]. (1998) 109:387–90. doi: 10.1016/S0924-980X(98)00035-6 9851294

[12] Piccione EA Sletten DM Staff NP Low PA. Autonomic system and amyotrophic lateral sclerosis. Muscle Nerve [Internet]. (2015) 51:676–9. doi: 10.1002/mus.24457 25211238 PMC4362936

[13] Atassi N Berry J Shui A Zach N Sherman A Sinani E. The PRO-ACT database. Neurol Int. (2014) 83:1719–25. doi: 10.1212/WNL.0000000000000951 PMC4239834 25298304

[14] Gibbons CH Schmidt P Biaggioni I Frazier-Mills C Freeman R Isaacson S. The recommendations of a consensus panel for the screening, diagnosis, and treatment of neurogenic orthostatic hypotension and associated supine hypertension. J Neurol. (2017) 264:1567–82. doi: 10.1007/s00415-016-8375-x 28050656 PMC5533816

[15] Fanciulli A Kerer K Leys F Seppi K Kaufmann H Norcliffe-Kaufmann L. Validation of the neurogenic orthostatic hypotension ratio with active standing. Ann Neurol. (2020) 88:643–5. doi: 10.1002/ana.25834 32596818

[16] Parkinson Progression Marker Initiative. The Parkinson progression marker initiative (PPMI). Prog Neurobiol. (2011) 95:629–35. 21930184 10.1016/j.pneurobio.2011.09.005 PMC9014725

[17] Finucane C O’Connell MDL Fan CW Savva GM Soraghan CJ Nolan H. Age-related normative changes in phasic orthostatic blood pressure in a large population study. Circulation. (2014) 130:1780–9. doi: 10.1161/CIRCULATIONAHA.114.009831 25278101

[18] Saedon NI Pin Tan M Frith J. The prevalence of orthostatic hypotension: a systematic review and Meta-analysis. J Gerontol Series A [Internet]. (2020) 75:117–22. doi: 10.1093/gerona/gly188 PMC6909901 30169579

[19] Méndez AS Melgarejo JD Mena LJ Chávez CA González AC Boggia J. Risk factors for orthostatic hypotension: differences between elderly men and women. Am J Hypertens [Internet]. (2018) 31:797–803. doi: 10.1093/ajh/hpy050 29617895 PMC7190879

[20] Coon EA Cutsforth-Gregory JK Benarroch EE. Neuropathology of autonomic dysfunction in synucleinopathies. Mov Disord [Internet]. (2018) 33:349–58. doi: 10.1002/mds.27186 29297596

[21] Xia H Suda S Bindom S Feng Y Gurley SB Seth D. ACE2-mediated reduction of oxidative stress in the central nervous system is associated with improvement of autonomic function. PLoS ONE [Internet]. (2011) 6:e22682. doi: 10.1371/journal.pone.0022682 21818366 PMC3144922

[22] Pisano F Miscio G Mazzuero G Lanfranchi P Colombo R Pinelli P. Decreased heart rate variability in amyotrophic lateral sclerosis. Muscle Nerve [Internet]. (1995) 18:1225–31. doi: 10.1002/mus.880181103 7565918

[23] Sachs C Conradi S Kaijser L. Autonomic function in amyotrophic lateral sclerosis: a study of cardiovascular responses. Acta Neurol Scand [Internet]. (2009) 71:373–8. doi: 10.1111/j.1600-0404.1985.tb03215.x 4013661

[24] Gibbons CH. Basics of autonomic nervous system function. Handb Clin Neurol. (2019) 160:407–18. doi: 10.1016/B978-0-444-64032-1.00027-8 31277865

[25] Fedorowski A Stavenow L Hedblad B Berglund G Nilsson PM Melander O. Consequences of orthostatic blood pressure variability in middle-aged men (the Malmö preventive project). J Hypertens [Internet]. (2010) 28:551–9. doi: 10.1097/HJH.0b013e3283350e8c 19952779

[26] Naschitz JE Rosner I. Orthostatic hypotension: framework of the syndrome. Postgrad Med J [Internet]. (2007) 83:568–74. doi: 10.1136/pgmj.2007.058198 17823222 PMC2600002

